# F-18 FDG PET/CT Findings of a Patient with Takayasu Arteritis Before and After Therapy

**DOI:** 10.4274/Mirt.021896

**Published:** 2012-04-01

**Authors:** Sait Sağer, Sabire Yılmaz, Meftune Özhan, Metin Halaç, Nurhan Ergül, Hediye Çiftci, T. Fikret Çermik

**Affiliations:** 1 Istanbul University Cerrahpaşa Medical Faculty, Department of Nuclear Medicine, Istanbul, Turkey; 2 Istanbul Education and Research Hospital, Clinic of Nuclear Medicine, Istanbul, Turkey

**Keywords:** Vasculitis, Takayasu arteritis, F-18 FDG

## Abstract

Vasculitis is defined as inflammation and necrosis with leukocytic infiltration of the blood vessel wall. Takayasu arteritis is a chronic inflammatory arteritis that primarily involves the aorta and its main branches. A 64-year-old female patient with a 2-month history of fever of unknown origin was presented to our clinic for F-18 FDG PET/CT imaging. Baseline PET/CT images demonstrated intense F-18 FDG uptake in the aorta, bilateral subclavian and brachiocephalic arteries consistent with Takayasu arteritis. After 2 months of immunosuppressive therapy, she was asymptomatic and follow-up FDG PET/CT scan showed almost complete disappearance of large vessels’ F-18 FDG uptake. FDG PET/CT is a sensitive technique for assessing presence of large-vessel vasculitis such as Takayasu arteritis, extent of large-vessel inflammation and disease activity after therapy.

**Conflict of interest:**None declared.

## INTRODUCTION

It is well known that radiolabeled glucose analogue F-18 fluoro-deoxyglucose (F-18 FDG) used in positron emission tomography (PET) accumulates in both malignant and inflammatory tissues (1). F-18 FDG accumulates in tumour cells but also in inflammatory tissues due to the over-expression of glucose transporter (mainly GLUT 1, 3) and overproduction of glycolytic enzymes (2). The large vessel vasculitis is considered to be the cause in 17% of all FUO (fever of unknown origin) patients (3). 

F-18 FDG PET/CT metabolic imaging is increasingly used in the investigation of patients with large vessel vasculitis. The accumulation of 18F-FDG delineates arterial wall inflammation in giant cell arteritis (GCA), Takayasu arteritis (TA) and reveals the extent of disease. F-18 FDG accumulation in the arterial wall reflects vasculitis activity and shows extent of the disease. F-18 FDG-PET is highly effective in detecting large-vessel vasculitis anywhere in the body and has high sensitivity and specificity (4). F-18 FDG PET/CT has a potential role to diagnose and monitor response to the therapy in large vessel vasculitis (5). 

In this case report, we aimed to show the role of F-18 FDG PET/CT imaging to assess the large-vessel vasculitis such as Takayasu arteritis (TA), extent of large-vessel inflammation and disease activity after the immunosuppressive therapy.

## CASE REPORT

**Figure 1:**
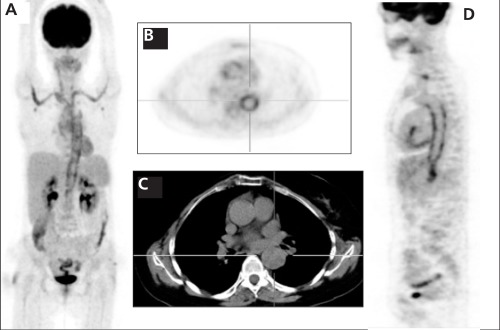
A 64-year-old woman with active Takayasu arteritis. Figure1A (MIP), Figure 1B (Axial PET), Figure 1D (Sagittal PET) imagesdemonstrated intense F 18 FDG uptake in the aorta, bilateral subclavianand brachiocephalic arteries consistent with Takayasu arteritis. The maximum standardized uptake value of the aorta was 9.2 beforetherapy

64-year-old female patient was admitted to our clinic for PET/CT examination because of fatigue, sweating, recurrent fever at 38-39 °C and weight loss. For PET/CT examination the patient was intravenously injected 510 MBq of F-18 FDG, after 6 hours of fasting state. After one hour of waiting period in a silent room, patient was imaged using an integrated PET/CT camera, which is consists of a 6-slice CT gantry integrated on a LSO based full ring PET scanner (Siemens Biograph 6, IL, Chicago USA). PET/CT images, Figure 1A (MIP), Figure 1B (Axial PET), Figure 1D (Sagittal PET) demonstrated intense FDG uptake in the aorta, bilateral subclavian and brachiocephalic arteries consistent with Takayasu arteritis. The maximum standardized uptake value of the aorta was 9.2 before therapy.

**Figure 2:**
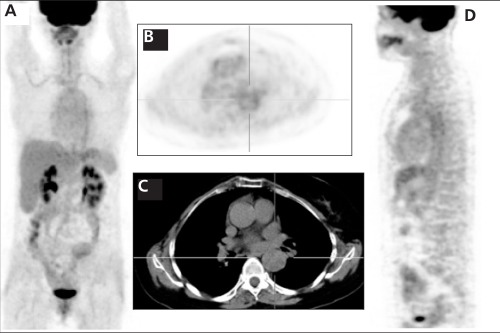
The same patients’ follow up F 18 FDG PET/CT images after2 months of immunosuppressive therapy. Figure 2A: MIP, 2B: AxialPET, 2D: Sagittal PET showed almost complete disappearance of largevessels’ F 18 FDG uptake but also minimal F 18 FDG uptake in theaorta and bilateral subclavian arteries. After therapy, the maximumstandardized uptake value of the aorta was 3.4. Figure 2C axial CTimages showed no pathological lesion or mass in the large vessels

Prednisolone and cyclophosphamide (110 mg/day) were started for immunosuppressive therapy. Two months after the therapy, follow-up PET/CT study (Figure 2A: MIP, 2B: Axial PET, 2D: Sagittal PET) showed almost complete disappearance of large vessels FDG uptake but also minimal FDG uptake in the aorta and bilateral subclavian arteries. Figure 1C and Figure 2C axial CT images showed no pathological lesion or mass in the large vessels. After therapy, the maximum standardized uptake value (SUVmax) of the aorta was 3.4. Patient’s clinical symptoms disappeared which is consistent with F-18 FDG PET/CT images. 

## LITERATURE REVIEW AND DISCUSSION

Several studies showed the value of F-18 FDG PET in the diagnosis of large vessel vasculitis via high F-18 FDG accumulation in the vascular lesions that could not be detected with other imaging techniques. Kobayashi et al. showed that intense F-18 FDG accumulation with a SUVmax of ≥2.7 in the vasculature of 2 of the 11 cases in the active stage of Takayasu arteritis and weak F-18 FDG accumulation with a SUVmax value of ≥2.3 in the other 9 active patients. They did not observe significant F-18 FDG accumulation in the patients with inactive disease (SUV≤1.2) and in 6 control healthy subjects (SUV < 1.3) (10). Webb M. et al. showed that achieved sensitivity of 92%, specificity of 100% and negative and positive predictive values of 85% and 100% respectively in the initial assessment of active vasculitis in TA (8). Andrews J. et al. showed that non-invasive imaging method F-18 FDG-PET imaging provided important additional information about disease activity when compared with x ray angiography (9). Vista E.G. et al. showed that three of four patients showed evidence of increased radiotracer uptake in the aorta consistent with TA (10). Kunihiko U. et al. observed specific accumulation of F-18 FDG-6-phosphate in the thoracic aorta and its direct branches leading to diagnose TA in a young woman in their case report (11). Schurgers M. et al. reported F-18 FDG-PET findings of a 24-year-old Caucasian woman presenting with fatigue, weight loss, a cardiac murmur, anemia and biochemical markers of inflammation due to TA (12).

Takayasu arteritis is a chronic inflammatory condition that affects the large blood vessels in the body mainly the aorta and its branches (6). The typical patient with Takayasu arteritis is a woman under the age of 40. Although the disease has a worldwide distribution, it appears to occur more often in Asian women. Its pathogenesis is still unknown. 

The diagnosis of Takayasu arteritis is diffucult. In most patients, the diagnosis is delayed until after the development of arterial injury. Radiological imaging studies might facilitate earlier diagnosis and have a role in monitoring disease progress; however, they remain limited in their ability to accurately quantify inflammatory disease activity. The disease activity can be estimated with general inflammatory markers such as C-reactive protein and erythrocyte sedimentation rate during treatment (13). However, acute phase reactants have shown to have poor sensitivity and specificity in confirming disease activity in TA patients.

F-18 FDG PET has been reported to be useful in the diagnosis of vascular diseases such as atherosclerosis and large vessel aortitis. It is highly effective in assessing the disease activity, extent of large-vessel inflammation and disease activity after therapy (14,15). F-18 FDG PET co-registered with CT can enhance the sensitivity of the 18F-FDG accumulation in the aorta, its branches, and the pulmonary artery due to Takayasu arteritis. In our case report, intense F-18 FDG uptake in the aorta, bilateral subclavian and brachiocephalic arteries are shown with PET/CT imaging in the initial assessment. It is known that inflammatory cells such as macrophages, fibroblasts, lymphocytes or neutrophils are shown to avidly take up F-18 FDG under active conditions (11). Although false negative results may occur during treatment or false positive results mainly occur in the differential diagnosis between vasculitis and atherosclerotic vessels in elderly patients, FDG-PET/CT has demonstrated the potential to non-invasively diagnose the onset of the vasculitis earlier than traditional anatomical imaging techniques (14). In our case, patients’ clinical symptoms have disappeared which is consistent with F-18 FDG PET/CT images after the therapy. Because of lower intensity in comparison to the baseline scan, consistent with persistent low-activity inflammation, patients’ therapy was continued.

## CONCLUSION

F-18 FDG PET/CT is a Nuclear Medicine imaging technique that provides the clinician with a non-invasive measure of disease activity in TA patients. Early diagnosis of active inflammation in TA patients provides timely treatment with appropriate therapy. The visual grading of vascular F-18 FDG uptake makes it possible to discriminate arteritis from active atherosclerosis.
